# RNAseq versus genome-predicted transcriptomes: a large population of novel transcripts identified in an Illumina-454 *Hydra* transcriptome

**DOI:** 10.1186/1471-2164-14-204

**Published:** 2013-03-25

**Authors:** Yvan Wenger, Brigitte Galliot

**Affiliations:** 1 Department of Genetics and Evolution, Institute of Genetics and Genomics in Geneva (iGE3), University of Geneva, Geneva, Switzerland

**Keywords:** Illumina and 454 RNAseq transcriptome assembly, *Hydra* transcriptome, Reciprocal Best Hits (RBH), Next generation sequencing, Genome-predicted transcriptome, Human orthologs in Hydra, Pseudogenes

## Abstract

**Background:**

Evolutionary studies benefit from deep sequencing technologies that generate genomic and transcriptomic sequences from a variety of organisms. Genome sequencing and RNAseq have complementary strengths. In this study, we present the assembly of the most complete *Hydra* transcriptome to date along with a comparative analysis of the specific features of RNAseq and genome-predicted transcriptomes currently available in the freshwater hydrozoan *Hydra vulgaris*.

**Results:**

To produce an accurate and extensive *Hydra* transcriptome, we combined Illumina and 454 Titanium reads, giving the primacy to Illumina over 454 reads to correct homopolymer errors. This strategy yielded an RNAseq transcriptome that contains 48’909 unique sequences including splice variants, representing approximately 24’450 distinct genes. Comparative analysis to the available genome-predicted transcriptomes identified 10’597 novel *Hydra* transcripts that encode 529 evolutionarily-conserved proteins. The annotation of 170 human orthologs points to critical functions in protein biosynthesis, FGF and TOR signaling, vesicle transport, immunity, cell cycle regulation, cell death, mitochondrial metabolism, transcription and chromatin regulation. However, a majority of these novel transcripts encodes short ORFs, at least 767 of them corresponding to pseudogenes. This RNAseq transcriptome also lacks 11’270 predicted transcripts that correspond either to silent genes or to genes expressed below the detection level of this study.

**Conclusions:**

We established a simple and powerful strategy to combine Illumina and 454 reads and we produced, with genome assistance, an extensive and accurate *Hydra* transcriptome. The comparative analysis of the RNAseq transcriptome with genome-predicted transcriptomes lead to the identification of large populations of novel as well as missing transcripts that might reflect *Hydra*-specific evolutionary events.

## Background

**Figure 1:**
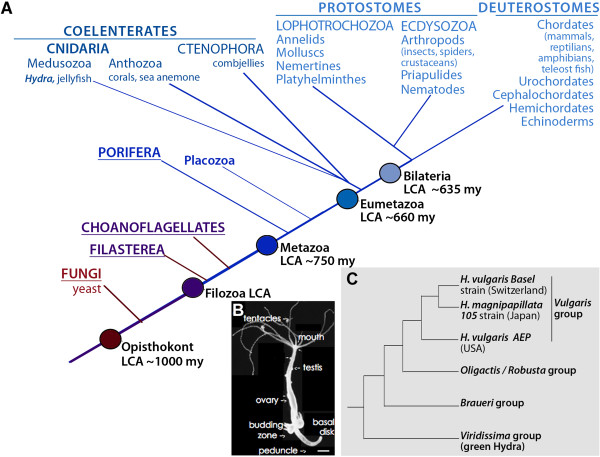
**Phylogenetic position of *Hydra*. A**) The phylogenetic tree of opisthokonts was drawn according to [12,13]. Together with ctenophores, cnidarians form the Coelenterate superphylum. Coelenterates together with bilaterians constitute Eumetazoa, whereas porifers (sponges) occupy a sister group position within Metazoa. Dates are expressed in million years (my) according to [14]. LCA: Last Common Ancestor. **B**) Anatomy of the adult *Hydra* polyp. Scale bar: 400 μm. **C**) Phylogenetic tree of the various *Hydra* species drawn according to [15,16]. Genome-predicted transcriptomes are available from *H*. *magnipapillata* (*Hm*) [3], a RNAseq-454 transcriptome was produced from *H*. *vulgaris AEP* (*AEP*) [11] and this study presents a RNAseq Illumina-454 transcriptome from *H*. *vulgaris Basel* (*Hv*).

*Hydra* is a freshwater polyp that belongs to Cnidaria, a sister group to Bilateria (Figure 1A) [1]. *Hydra* anatomy is organized as a tube with an oral-aboral axis consisting of two cell-layers and three populations of stem cells (Figure 1B). Since the discovery of *Hydra* regeneration in the mid XVIII^th^ century, *Hydra* provides a unique model system to study how exogenous perturbations can reactivate a developmental program in an adult organism (see in [2]). Indeed, *Hydra* possesses the amazing ability to regenerate any missing part upon bisection of its body column. To dissect the genetic cascades supporting regenerative processes, a complete report of the genetic toolkit expressed in this animal is necessary. Among cnidarians, genomic data are currently available from three species, *Hydra magnipapillata*[3], *Nematostella vectensis* (sea anemone) [4] and *Acropora digitifera* (coral) [5]. Transcriptomic data are available from the colonial hydroid *Hydractinia equinata*[6], from the jellyfish *Clytia haemispherica*[7], from the coral *Acropora millepora*[8,9] and from two distinct *Hydra* strains that belong to the heterogeneous *vulgaris* group [10,11]. In addition, two sets of putative transcripts, called here pred-CA and pred-RP, have been predicted from the *Hydra magnipapillata* genome [3]. In spite of these efforts, the transcriptome of *Hydra* is still incomplete.

To produce a *Hydra* transcriptome that would account for a high proportion of full-length RNA sequences, we combined two widely used high-throughput sequencing pipelines, developed by Illumina [17] and 454 Life Sciences [18] respectively. The Illumina technology produces shorter reads (currently up to 150 bp) at a lower cost per base than the longer reads (~350 bp) produced by the Roche 454 Titanium technology. Beside these differences, the two technologies differ by the type of errors they generate, mostly base substitutions in Illumina, and micro-insertions or deletions in 454 homopolymer stretches [19], although the overall error rate is much lower in assemblies generated using Illumina reads, partly due to higher coverages [19]. Consequently assemblies of 454 RNAseq reads frequently contain frameshift errors, which lead to truncated proteins after conceptual translation. Despite sustained progress in the field, no single standard assembly procedure mixing reads of different technologies has yet met general agreement. Here, we reasoned that whenever Illumina and 454 sequences corresponding to a transcript were available, Illumina-derived contigs (containing much fewer homopolymer errors [19]) were to be selected in priority for building consensus stretches. To reflect this choice, we adapted a method that was previously used to assemble Illumina contigs to 454 reads [20]. Thanks to this strategy, we produced a *Hydra* transcriptome that contains 48’909 unique transcripts, including 10’597 novel sequences. Then, we performed a systematic comparative analysis of the RNAseq and genome-predicted transcriptomes.

## Results

### Production of an extensive transcriptome from Illumina and 454 reads

**Figure 2:**
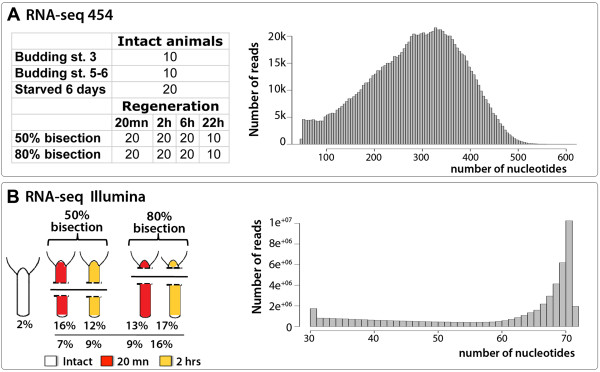
**Scheme representing the conditions and the reads obtained from *Hydra vulgaris *for Illumina and 454 RNAseq. A**) Left panel: number of intact and regenerating *Hydra* mixed to extract mRNA used in 454 sequencing. Right panel: length distribution of 454 reads after adapter trimming. **B**) Left panel: percentages of mRNA taken from *Hydra* in intact and regenerative conditions, as deduced from their respective number of reads sequenced. Right panel: length distribution of Illumina reads after barcode removal and adapter trimming.

We produced a *Hydra* RNAseq transcriptome by combining 454 and Illumina reads obtained from the *Hydra vulgaris* strain “Basel”. This European strain is very closely related to the Japanese strain *Hydra magnipapillata*[15,16], and they both belong to the *vulgaris* group (Figure 1C). In fact, those two strains are hardly differentiable at the molecular level and are distinguished based on their geographical origin [16]. The comparison of the *Hydra vulgaris* “Basel” sequence data produced in this study to the *Hydra magnipapillata* genomic sequences [3] typically resulted in 1% or less differences at the nucleotide level (see Additional file 1: Figure S2B). To increase the odds of capturing gene specifically expressed during regeneration for subsequent studies, cDNAs libraries were prepared mostly from regenerating *Hydra* (Figure 2AB). The size distribution of the 454 and Illumina reads that we obtained is shown in Figure 2A, 2B, respectively.

**Figure 3:**
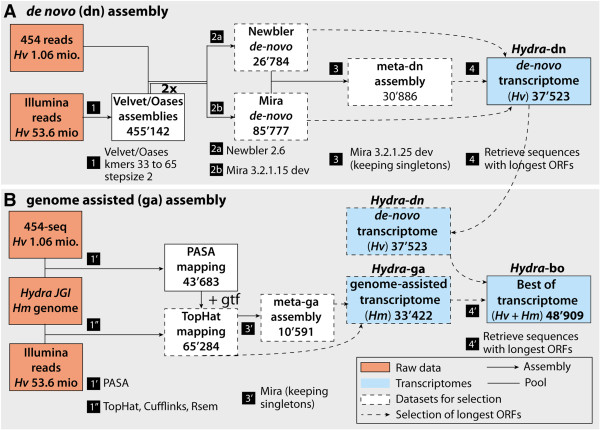
**Assembly procedures of the *de*-*novo *(*dn*) and genome-assisted (ga) *Hydra *transcriptomes. A, B**) Schemes showing the *de*-*novo* (*dn*) assembly process of *Hydra* RNAseq reads (**A**), and the genome-assisted (ga) and “best of” (bo) assembly processes of *Hydra* transcriptomes (**B**). Numbers of sequences are indicated at each step. For steps 2a and 2b, artificial Phred quality values were produced for the dataset called “Contigs” and the sequences were duplicated in order to further correct frameshifts due to 454 sequencing (see Methods section for details).

To increase the strength of the Illumina contigs against the 454 reads, we first performed an initial assembly of the Illumina reads into contigs using Velvet/Oases (Figure 3A). Then in the course of the subsequent assemblies, we performed the following manipulations (see Methods for details): 1) Illumina contigs were duplicated (Additional file 1: Figure S1); 2) artificial base qualities were generated. Finally, contigs longer than 2000 nt were chopped into 1999 nt with 1899 nt overlapping segments to accommodate a requirement of the Newbler assembler. The resulting sequences were used as input for Mira (Mira-*dn*) and Newbler (*Newbler-dn*) assemblies along with 454 reads (Figure 3A).

The outputs of Mira-*dn* and Newbler-*dn* were then joined using a second round of Mira assembly with similar parameters (the assembly is called meta-*dn*). Occasionally, meta-*dn* contained longer coding sequences than the two original datasets and those sequences were retained (see Methods for details). The *de*-*novo* assembly was complemented by a genome-assisted (ga) assembly (Figure 3B). With the ga procedure, and in contrast to the *de*-*novo* procedure, very short overlaps (up to 1 nt) between RNAseq reads that can be mapped unambiguously to the genome are sufficient to expect contiguity and assemble reads into contigs. Thus, in the case of transcripts with low coverage, the ga assembly procedure frequently leads to more contiguous sequences when the transcribed regions are available from the genome.

The ga assembly consisted in the independent mapping of the 454 and the Illumina reads to the genome. The cufflinks software [21] was then used to reunite the results of both mappings in a single summary file, and the sequences of the modeled transcripts were extracted (see Methods for details). Similarly to the *de-novo* assembly process, a second round of assembly was performed using Mira with this dataset (meta-ga assembly) and the longest open reading frames (ORFs) were retrieved for each genome-assisted sequence (*Hydra-ga*). Finally, a dataset reuniting the sequences with the longest ORFs that were found among the *de-novo* or genome-assisted assemblies was retrieved (named *Hydra*-bestof-*Hydra*-bo-). This *Hydra*-bo dataset contains 48’909 sequences, 45’269 of which are longer than 200 nt and have been deposited at the European Nucleotide Archive (ENA) under the accessions HAAC01000001-HAAC01045269.

### Production of an hybrid RNAseq/genome-predicted transcriptome (Hydra-meta) and saturation analysis

**Figure 4:**
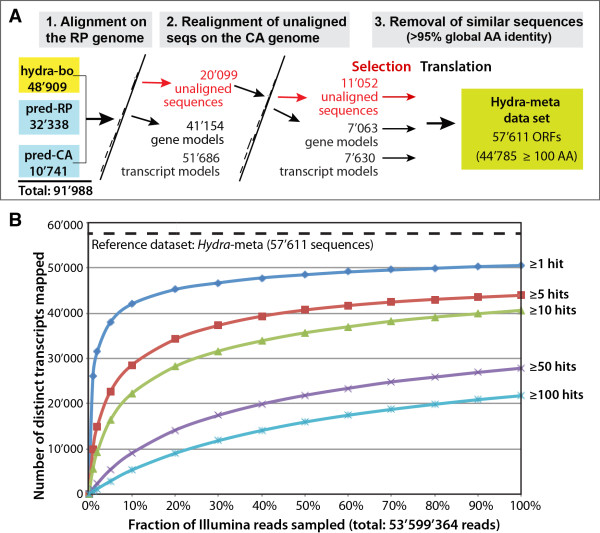
**Evaluation of the completeness of the *Hydra***-**bo transcriptome. A**) Construction of the *Hydra*-meta transcriptome, which contains an unredundant assembly of the *Hydra*-bo and predicted sequences (pred-CA and pred-RP). **B**) Saturation analysis of transcripts representation (y-axis) with increasing numbers of sampled reads (x-axis). The *Hydra*-meta transcriptome was used as a reference. Subsets of randomly sampled Illumina reads were collected and mapped to the reference using Bowtie. The numbers of mapped reads (hits) were counted for each reference sequence.

To evaluate the complexity present in the Illumina reads against a comprehensive *Hydra* transcriptome that might be used as a reference for further studies, the *Hydra*-bo transcriptome was pooled with genome-predicted datasets (Figure 4A, see Methods for details), resulting in a non-redundant set of contigs that contains a mixture between RNAseq and genome-predicted sequences (*Hydra*-meta). Subsets of randomly chosen Illumina reads were mapped onto the *Hydra*-meta dataset and the number of reads mapped to each sequence was counted (Figure 4B). Of the 57’611 sequences present in the *Hydra*-meta dataset, 7’070 were never mapped with an Illumina read, pointing to insufficient Illumina sequencing depth, or genes not expressed in the condition tested. Another possibility is that some of the predicted sequences are a consequence of overprediction and are in facts never expressed although this cannot be determined from our data. The number of sequences mapped by 1 or more reads (50’541 sequences) exhibited saturation, as a random subset containing 20% of all Illumina reads was sufficient to target 89.5% of them. However, even if a large fraction of transcripts appeared to be detected in this study, the fact that only 21’746 contigs are mapped with 100 or more reads indicates that the sequencing depth of the Illumina dataset alone (53.6M reads) might be insufficient to assemble full length contigs for weakly expressed transcripts.

### Gene number estimation

**Figure 5:**
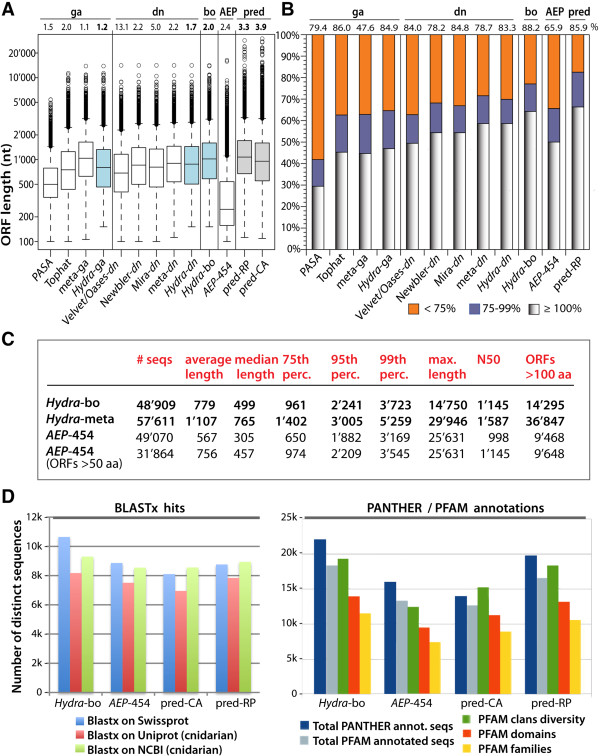
**Comparative analysis of the *Hydra vulgaris *(Illumina-454 RNAseq), *Hydra AEP *(454 RNAseq) and *Hydra magnipapillata *(genome-predicted) transcriptomes. A**) Boxplot representing the ORF lengths (nucleotides) of the intermediary (white) and final (blue) assemblies of the genome-assisted (*Hydra-dn*) and “best of” (*Hydra*-bo) transcriptomes. For comparison see the distribution of ORF lengths from the *AEP*-*454* (white) [11] and predicted (pred-RP, pred-CA, grey) [3] transcriptomes. Open circles represent outliers. Horizontal bars represent, from bottom to top, minimum, lower quartile, median, upper quartile, and maximum ORF lengths (excluding outliers). Numbers at the top indicate redundancy indexes. **B**) Comparison of the sizes of the coding sequences between the datasets shown in A and the pred-CA transcriptome. The pred-CA coding sequences were aligned against each sequence of every other dataset using BlastN+ without low complexity filter. First hits were retained if the alignment was uninterrupted for more than 100 nt with at least 95% sequence identity. The sizes of the matched and queried sequences were compared and classified into three classes according to the size of the tested sequence (hit): ≥ 100% if larger or equal to the size of the corresponding pred-CA sequence (greyish shadow), between 99% and 75% (blue), lower than 75% (orange). Top numbers indicate the percentage of pred-CA sequences matched by the transcriptome indicated on the x-axis. **C**) Characteristics of the *Hydra*-bo, *Hydra*-meta and *AEP*-*454* RNAseq transcriptomes. As *Hydra*-bo and *Hydra*-meta contain exclusively sequences that are at least 150 coding nucleotides long, the same criteria was applied to the *AEP*-*454* dataset. The last column indicates the number of full-length (start and stop codons) ORFs longer than 100 AAs. **D**) Number of functionally annotated sequences in the RNAseq and genome-predicted transcriptomes when analyzed with BlastX+ (left), Pfam or Panther (right).

A rough estimate of the number of *Hydra* genes based on transcripts only was deduced from the number of sequences in each dataset associated with a measure of the redundancies they contain. Indeed, the presence of multiple similar sequences in a dataset, due for example to allelic or splice variants, would lead to an overestimation of the number of genes if not corrected for internal redundancy. To assess the presence of multiple near-identical sequences within transcriptomes, we aligned the coding nucleotide sequences from each dataset against its whole dataset using megablast [22]. The number of hits retrieved by each query sequence was counted and a single value representing the average number of matches per query was calculated. The measure, called the redundancy index (RI), is an indication of the redundancy of the considered transcriptome (see in Figure 5A the numbers at the top of each lane. See Methods).

*Hydra*-bo sequences retrieved 2.0 matches on average, a RI value significantly lower than those obtained for the genome-predicted transcriptomes (3.3 and 3.9 for pred-CA and pred-RP respectively, Figure 5A, top numbers). As each *Hydra*-bo sequence matches two closely related sequences on the same set on average, we estimate the number of *Hydra* genes to be ~24’450 (48’909/2.0). Following the same calculation rules, the number of genes fall to 4’549 and 9’799 for the genome-predicted pred-CA and pred-RP transcriptomes and reaches 19’628 with the *AEP*-*454* transcriptome [11].

An independent estimate of the number of genes represented in the *Hydra*-bo, pred-CA, and pred-RP, was obtained by mapping their sequences back to the genome and counting the number of expressed genomic loci. Of the three datasets, pred-CA yielded the lowest number with 16’302 estimated genes, and the pred-RP dataset had the highest estimate with 31’243 genes. For the *Hydra*-bo transcriptome, we counted a total of 28’520 different expressed loci. Both numbers, 24’455 based on transcript redundancy or 28’520 based on genetic loci exceed the number previously predicted to be ~20’000 [3]. However this initial number did not include pseudogenes or transposons. Therefore the 24’500 to 28’500 estimation is compatible with the fact that the RNAseq transcriptome assembled here incorporates a large share of all sequences expressed in *Hydra*.

### Comparative analysis between the Hydra-bo, AEP-454, and genome-predicted transcriptomes

To compare the existing datasets, we first assessed their physical characteristics, and next evaluated their respective functional content. The lengths of the coding sequences of the *Hydra*-bo, pred-CA and pred-RP transcriptomes exhibit similar size distribution (Figure 5A) except for a few sequences from the genome-predicted sets that appear longer. By contrast the *AEP*-*454* transcriptome contains a large proportion of small ORFs (Figure 5A, 5C). The lengths of the coding sequences of each dataset were then compared to the length of the corresponding coding sequences found in the pred-CA transcriptome (thus using each of the pred-CA sequences as a standard, see Methods for details). This analysis shows a progressive increase in the length of the successive intermediary datasets, with the first assembly (PASA) containing the shortest coding sequences and the final assembly (*Hydra*-bo) containing the longest (Figure 5B). Over 60% of *Hydra*-bo sequences display either equal or longer coding length when compared to pred-CA sequences (Figure 5B, grey shadow bar). Finally, the *Hydra*-bo transcriptome contained more sequence representatives of pred-CA sequences than any other dataset (88.2%, Figure 5B, top percentages). In comparison, the *AEP*-*454* dataset captured 65.9% of the 32’338 pred-CA sequences. The number of full length ORFs (containing a start and a stop codon) longer than 100 amino acids (AA) was 14’295 for *Hydra*-bo and 9’648 for *AEP*-*454* (Figure 5C). In contrast, the *Hydra*-meta dataset contains a much larger number of full length ORFs (36’847).

To compare the content of the *Hydra*-bo, *AEP*-*454*, and predicted transcriptomes, Blasts and functional annotations using Pfam and Panther were performed (Figure 5D). Overall, *Hydra*-bo matched more sequences (Figure 5D, left panel) and encoded more functional families than the three other transcriptomes tested here (Figure 5D, right panel). These gene families are listed in Additional file 2. We conclude that *Hydra*-bo not only contains longer sequences than *AEP*-*454*, but also encodes a greater diversity of transcripts when compared to *AEP*-*454* and genome-predicted transcriptomes.

### Unpredicted sequences in the *Hydra*-bo RNAseq transcriptome

**Figure 6:**
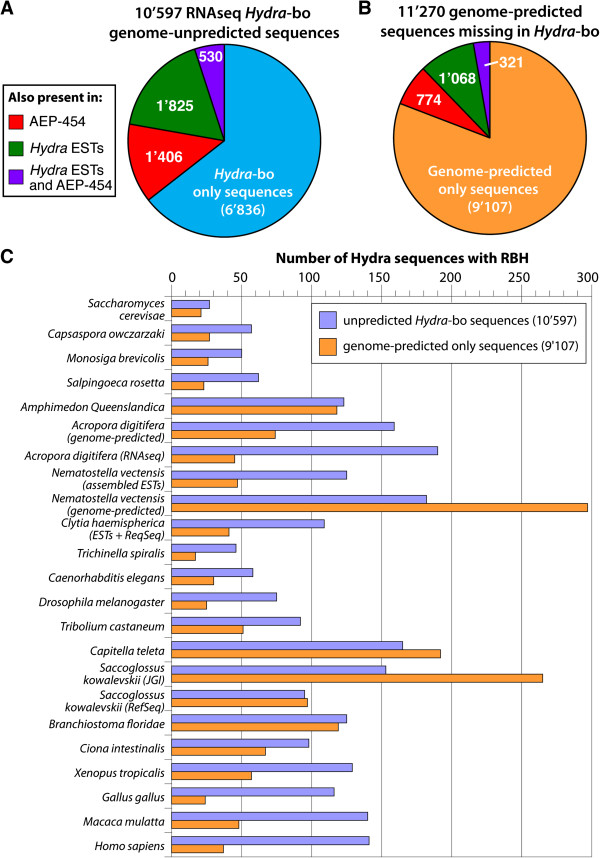
**Comparative analysis of the RNAseq only and predicted-only sequences. A**) Pie chart showing the distribution of the 10’597 *Hydra*-bo sequences not found in the genome-predicted transcriptomes, thus named “unpredicted”: 3’761 align to *Hydra* ESTs and/or to sequences from the *AEP*-*454* transcriptome whereas 6’836 appear novel. **B**) Pie chart showing the distribution of the 11’270 genome-predicted sequences that were not found in the *Hydra*-bo dataset: 2’163 align to *Hydra* ESTs and/or to *AEP*-*454* sequences but 9’107 sequences do not align to any expressed transcripts, thus named “predicted-only”. **C**) Number of Reciprocal Best Hits (RBHs) identified among predicted-only (9’107) and unpredicted (10’597) sequences when tested on a variety of opisthokont species. RBHs occurrence was assessed for *Hydra*-bo and predicted transcriptomes in each species individually, using whole proteomes and whole deduced proteomes. Predicted-only and unpredicted sequences corresponding to RBH orthologs were then extracted.

To identify the transcripts present in *Hydra*-bo but absent from the two sets of genome-predicted sequences, we aligned the *Hydra*-bo sequences to a pooled version of the predicted datasets. Queries without hits (95% similarity threshold on nucleotides, without using low complexity masking) were retrieved and counted (Figure 6). Among *Hydra*-bo sequences, we found 38’168 (78%) sequences that were common between the predicted and the RNAseq datasets but also 10’597 *Hydra*-bo sequences that were missing in the genome-predicted transcriptomes. The expression of a large fraction of these “RNAseq only” sequences was actually confirmed (Figure 6A), 2’355 in ESTs, 1’936 in the *AEP*-*454* transcriptome. As a consequence, 6’836 RNAseq-only sequences remain completely novel. In a similar way, we found 11’270 genome-predicted sequences missing in *Hydra*-bo. Among these “predicted-only” sequences, 1’389 were identified in ESTs, 1’094 in the *AEP*-*454* transcriptome, leaving 9’107 sequences unique to the genome-predicted sets (Figure 6B). All these sequences exhibit a low GC content (Additional file 1: Figure S2A) that is typical for *Hydra*.

To verify whether the 10’597 RNAseq-only transcripts are indeed corresponding to *Hydra* genes, we aligned them to genomic sequences allowing for a greater level of mismatches than in the previous analysis (i.e. without using the 95% identity threshold) (Additional file 1: Figure S2B). 9’884 sequences (93%) aligned to the genomic sequences with more than 75% identity. The 10’597 RNAseq only transcripts were then aligned to proteins deduced from the full genome-predicted transcriptomes using BlastX+ (Additional file 1: Figure S2C). 4’170 (39.5%) transcripts were reliably mapped to genome-predicted proteins (E-value <10^-10^). The protein-alignments conservation ranged from 20% to 100% with an average of 65.3%, indicating that some RNAseq only transcripts probably correspond to paralogs of predicted sequences, whereas a large number of them share only low sequence identity and thus likely encode proteins not represented in the predicted datasets.

Finally, we compared the length of the ORFs of the RNAseq only and predicted only datasets (Additional file 1: Figure S2D). We noted a significant difference as the RNAseq only set contains shorter coding sequences with only 3’494 (33.0%) encoding proteins longer than 100 amino acids versus 8’115 (89.4%) in the genome-predicted only set. In summary, both datasets of transcripts seem to correspond to functional genes that encode *Hydra* proteins although with striking differences in terms of ORF length.

### Novel *Hydra*-human orthologs identified among the RNAseq only sequences

To characterize the proteins encoded by the RNAseq only and predicted-only sequences, we performed Reciprocal best hits (RBHs), a procedure that provides a powerful and reliable method to identify orthologs among large and multiple sequence populations [23]. As a first step, we deduced the proteomes of the *Hydra*-bo, *Hydra*-meta, *AEP*-*454* and genome-predicted transcriptomes, producing four distinct whole *Hydra* proteomes. Next, we tested these four *Hydra* proteomes against the proteomes of 20 species representative of non-metazoan (fungi, filifera, choanoflagellates), basal metazoan (porifers), basal eumetazoan (cnidarians) as well as bilaterian (lophotrochozoans, ecdysozoans, deuterostomes) phyla (Additional file 1: Figure S3). We detected a similar numbers of pairwise orthologs in the *Hydra*-bo, the *Hydra*-meta and the genome-predicted proteomes, although slightly higher in the *Hydra*-meta dataset. By contrast the number of orthologs retrieved from the *AEP*-*454* proteome was consistently lower in all species as anticipated from previous analyses.

**Figure 7:**
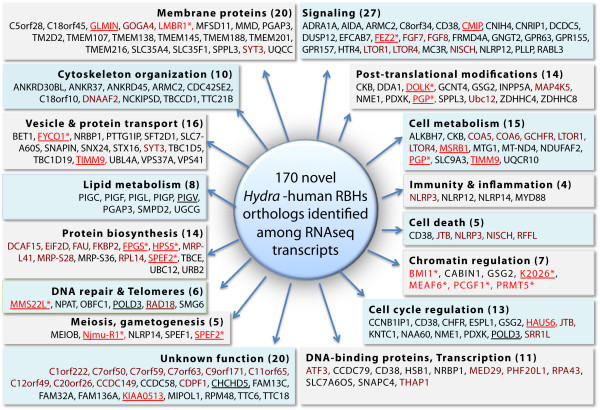
***Hydra *RNAseq only genes with RBHs on human orthologs.** 170 *Hydra* proteins were selected after RBHs with the human proteome (E-value <10^-8^). The names and predicted functions of these proteins were deduced from the annotation of the corresponding human othologs (Additional file 3). The phylogenetic relationships were tested for 14 of them (written red with asterisk) and confirmed in all cases (Additional file 1: Figure S4). Underlined names correspond to genes whose expression was tested in RT-PCR (Additional file 1: Figure S5A), confirmed in all cases, cloned and sequenced.

Similarly we retrieved the RBH orthologs present in the RNAseq only and predicted-only datasets from RBH computed on whole proteomes (as described in the previous paragraph). In both datasets, this analysis identified low numbers of orthologous proteins, 809 (8.9%) among the predicted-only sequences and 529 (5.0%) in the RNAseq only dataset (Figure 6C). This result suggests that a large fraction of these two gene datasets encode *Hydra*-specific proteins, and might thus be considered as taxon-restricted genes [7]. Nevertheless, we identified 170 novel orthologs to human proteins among novel *Hydra* sequences (Figure 7). The annotations of the human proteins point to a variety of molecular and cellular functions, encompassing cellular signaling (27) including RBH-orthologs to two distinct FGF proteins, but also membrane proteins (20), cell metabolism (15) including RBH-orthologs to LTOR1, LTOR4, to cytochrome c oxidase assembly proteins 5 and 6 (-COA5, COA6-), post-translational modifications (14), vesicle and protein transport (16) including a RBH-ortholog to synaptotagmin, cell cycle regulation (13), protein biosynthesis (14), DNA-binding activity and transcription (11) including RBH-orthologs to ATF3, RPA43, THAP1, cytoskeletal organization (10), lipid metabolism (8), chromatin regulation (7 see below), cell death (5), DNA repair and telomeres (6), meiosis and gametogenesis (5), immunity and inflammation (4) including RBH-orthologs to NLRP3, NLRP12, NLRP14 but also 20 proteins with unknown functions in human (complete sequences listed in Additional file 3).

The phylogenetic relationships obtained by RBH were verified for 14 proteins, including the chromatin regulators BMI1, MEAF6, PCGF1, PRMT5, K2026, the kinase DOLK, the phosphatase PGP, the mitochondrial synthase FPGS, and orthology was confirmed for all of them (see phylogenetic trees in Additional file 1: Figure S4). Finally we checked that these novel genes are indeed expressed in *Hydra*. We randomly picked a subset of them (18), successfully amplified all of them from cDNA (Additional file 1: Figure S5) and confirmed by cloning and sequencing their nucleotide sequence (Additional file 4). Hence the RNAseq only dataset contains a large number of expressed genes thus far never identified. Some of them are conserved between evolutionarily-distant species, and the human annotation suggests a functional role in a variety of molecular and cellular processes.

#### A large population of unpredicted transcripts with short ORFs and pseudogenes

**Figure 8:**
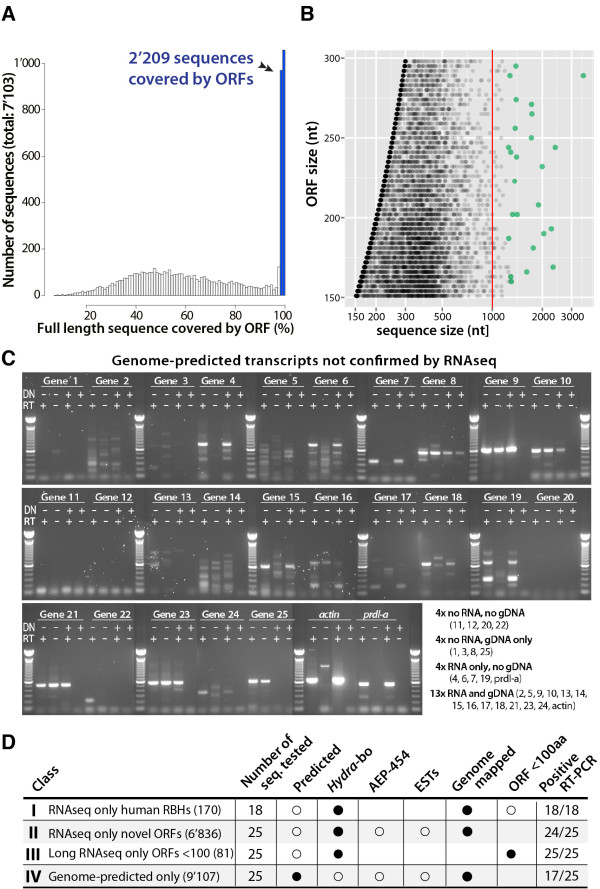
**Expression and phylogenetic analyses of the genome-unpredicted RNAseq only novel *Hydra *gene families. A**) Graph showing the proportion of the sequence covered by the ORF among the genome-unpredicted 7’103 RNAseq transcripts that encode short ORFs (<100 AA). 2’209 (31%) transcripts encode ORFs that cover >98% of their full length. **B**) Nucleotide sequence length of the transcripts analyzed in A: 81 are larger than 1’000 nt long (red line). The 25 largest contigs were tested by RT-PCR and all were successfully amplified (green dots). See Additional file 1: Figure S5. **C**) RT-PCR analysis of the expression of 25 genome-predicted *Hydra* transcripts not confirmed by RNAseq. To distinguish between gene and transcript amplification, PCR were performed on total RNA treated (+) or not (-) with DNAse (DN) and reverse transcribed (RT+) or not (RT-). **D**) The RNAseq only genes (i.e. genome-unpredicted), were sorted into three classes according to the presence (full dot) or the absence (empty dot) of the criteria specified above the first row. The expression of 18 sequences of class I and 25 sequences of classes II, III and IV was tested by RT-PCR.

From the 10’597 RNAseq only contigs, 7’103 encode putative ORFs shorter than 100 residues with an average length of 212 coding nt. The profile of the fraction of their coding lengths over their total length shows that 2’209 of these contigs have an ORF spanning over ≥95% of their length (Figure 8A). The vast majority of these contigs actually correspond to truncated fragments of longer coding units, which were nevertheless not described so far (based on sequence comparison using an independent transcriptome assembly with deeper coverage, Wanda Buzgariu, unpublished). To confirm the expression of RNAseq only sequences, 25 RNAseq transcripts never identified in any other dataset were selected and all were successfully amplified by RT-PCR (Figure 8D, Additional file 1: Figure S5, and Additional file 4).

Beside these short transcripts encoding short ORFs, we also noticed a subset of long contigs that encode short ORFs. Indeed we found 81 transcripts that extend from 1000 bp up to 3’531 bp but encode ORFs shorter than 100 residues (Figure 8B). We tested the expression of the 25 longest of these contigs by RT-PCR and 24 out of the 25 were successfully amplified (Figure 8D, and Additional file 1: Figure S5 and Additional file 4). These transcripts might correspond to long non-coding transcripts (ncRNA) although typical secondary structures found in ncRNAs could not be detected (data not shown). However, when these sequences were tested using BlastX+, we found that 18 of them hit on several locations a unique long protein sequence most often from *Hydra*, but also from *Nematostella* or even from bilaterian species (Additional file 2). This result suggested that these long contigs with short ORFs actually correspond to proteins fragmented by numerous mutations leading to stop codons. As these contigs align perfectly on the genome, we could exclude errors during the assembly process. We then searched systematically for this type of RNAseq only transcripts, i.e. that would satisfy both criteria, a perfect match on genomic sequences and no good match with genome-predicted transcripts (<95% sequence identity), indicating that they were arising from other loci. On the 4'894 RNAseq-only with short ORFs that do not span their full length, 3'958 sequences perfectly align to the genome (100% identity and no gaps). 767 of those align to predicted sequences with less than 95% sequence identity. This number of 767 pseudogenes is likely an underestimate given the stringency of the criteria used here.

### Genome-predicted genes can be active or silent

To test the expression of genome-predicted sequences, we selected 25 genome-predicted transcripts that fulfilled the following criteria: not represented in any transcript dataset (*Hydra*-bo, *AEP*-*454*, ESTs), but matching a jellyfish ortholog that would be expressed among the *Clytia* EST dataset (Additional file 4). The RT-PCR experiment was performed in conditions that ruled out any genomic contamination, i.e. RNA treated with DNAse or not, PCR amplification performed on RNA and on cDNA (Figure 8C). Among the 25 genome-predicted only sequences tested that way, we could convincingly amplify 17 of them (Figure 8C), indicating that a large proportion of these genome-predicted only sequences are indeed expressed in homeostatic and/or regenerating conditions in *Hydra* polyps. But this result also points to the 8/25 sequences that could not be amplified, and thus are expressed at a very low level or not expressed at all in adult, budding or regenerating *Hydra* polyps. This result would need confirmation at a larger scale but if confirmed would allow screening strategies where genes active in jellyfish and silent in *Hydra* could be identified. Such approaches could for example be useful to help characterizing genes involved in the aplanulata transition, i.e. when hydrozoan embryos lost the planula stage and became direct developpers, when hydrozoa polyps lost the potential for budding medusae and thus dramatically modified their life cycle.

## Discussion

### A powerful strategy to generate extensive and accurate transcriptomes

This study describes novel strategies to combine two widely used high-throughput sequencing technologies, Illumina and 454 RNAseq, in order to compensate for their specific errors and to produce accurate transcriptomes. Here we applied these strategies to RNAseq transcripts of the freshwater cnidarian *Hydra* resulting from the assembly of 53.6 millions Illumina short reads to 1.2 million 454 Titanium longer reads. As each assembly has its own specificities, in particular with respect to homopolymer errors, the sequences from several datasets were pooled, and the sequences with the longest ORFs among nearly identical transcripts were selected. This selection ensured that duplicated sequences, either partial or containing frameshifts were discarded from the dataset when a longer sequence was present. This study shows that the combination of different RNAseq methodologies provides an efficient and cost-effective strategy for further characterizing gene diversity in species where a finished genome is not available. In particular, homopolymer errors were effectively suppressed by our strategy. In turn, in giving priority to Illumina over 454 sequences, it is possible that some substitution-type Illumina sequencing errors are introduced although Illumina contigs generally exhibit lower substitution errors than 454 ones as well [19].

The RNA-seq transcriptome we established for the freshwater cnidarian *Hydra* fulfills several criteria for high fidelity when compared to other published transcriptomes, as evidenced by the different criteria used to compare them; namely the total number of transcripts including the presence of a large batch of novel transcripts, the length of these transcripts and their redundancy. These characteristics will be helpful to study the regulation of alternative splicing in developmental and stress contexts [24]. Finally, we took advantage of the genomic-derived sequences to extend and complete the RNAseq transcriptome. We now estimate that the *Hydra* transcriptome contains approximately 24’450 unique genes.

### As novelties, genes encoding evolutionarily-conserved proteins and short ORFs in Hydra

As a result of the comparative analysis we conducted between the genome-predicted transcriptomes [3], the *AEP*-*454* RNAseq transcriptome [11] and the *Hydra*-bo RNAseq transcriptome reported here, we identified 10’597 novel sequences in the *Hydra vulgaris* RNAseq transcriptome, most of them actually unpredicted but present in genomic contigs, and 35.5% found expressed either in the *AEP*-*454* transcriptome and/or in ESTs. The RBH analysis of these RNAseq only sequences identified 529 evolutionarily conserved proteins with at least one ortholog among the other tested species. 170 of these sequences show orthology with human proteins annotated for molecular and cellular functions that can now be tested in *Hydra*. Some protein-coding genes missing in the current version of the *Hydra* genome can be explained by its fragmented status, which prevents the systematic prediction of the transcriptional units. However, and quite surprisingly, a majority of these novel sequences encode short ORFs (7’103 contain ORFs <100 residues), which could not be predicted. A fraction of these sequences possibly correspond to peptide coding units as peptides are abundant in *Hydra*[25], whereas some longer transcripts seem to be expressed as pseudogenes. This result is of interest as pseudogenes are known to be submitted to gene regulation and to regulate themselves the level of expression of coding genes through a variety of mechanisms [26].

## Conclusions

The RNAseq transcriptome produced in this study together with the genome-predicted transcriptomes provides a solid reference for further molecular studies using *Hydra* as a model system. Within Cnidaria, this *Hydra* transcriptome together with the cnidarian transcriptomic and genomic sequences now available from *Hydra*[3,11,27], *Hydractinia*[6], *Clytia*[7], *Nematostella*[4] and *Acropora*[5,8,9] will help clarify the genetic sorting process that occurred along the evolution of medusozoans and anthozoans, and thus provide a clearer picture of the genetic equipment of the last common ancestor of cnidarians. Moreover, the classical *Hydra* model system is nowadays used in a variety of research topics such as regeneration, developmental processes, aging, stem cell biology, innate immunity and ecotoxicology. The deep comparison between genome-predicted and RNAseq transcriptomes offers new tools for phylogenetic reconstruction and should help decipher the gene regulatory networks involved in these processes. This econstitution is essential to highlight the outlines of the molecular pathways and biological processes at work in tissues of bilaterian ancestors.

## Methods

### *Hydra* culture and regeneration conditions

*Hydra vulgaris* (*Hv*, Basel strain) were cultured as described in [28] and fed 3x a week with freshly hatched *Artemia*. All animals were starved for at least three days before RNA extraction. For 454 sequencing, animals were starved for 3 or 6 days depending on the condition (Figure 2A). For Illumina sequencing, beside 100 intact animals, 200 budless animals per condition were bisected either at 80% (decapitation) or 50% (mid-gastric bisection) of the body length as indicated in Figure 2B. Upper and lower halves were then separately collected either between 10 and 20 min or 2 hours after bisection and transferred in RNALater (Ambion) prior to RNA extraction.

### Preparation and assembly of the 454 reads

For 454 sequencing, intact and regenerating *Hydra* were pooled prior to RNA extraction (RNAeasy kit, Qiagen). After DNase treatment (NucleoSpin RNA II kit, Macherey & Nagel), the polyA fraction was selected (Dynabeads Oligo(dT)25, Invitrogen) and a normalized random-primed cDNA was prepared and sequenced by GATC Biotech (Konstanz). Briefly, first-strand cDNA synthesis was primed with a N6 randomized primer (biomers.net), standard 454 adapters were ligated to the 5' and 3' ends of the cDNA and the cDNA was finally amplified with 10 PCR cycles using the Herculase II (Agilent Technologies) proof reading enzyme. Normalization was carried out by performing one cycle of denaturation and re-annealing of the cDNA, removal of the double-stranded cDNA by passing the mixture over a hydroxylapatite column, and amplification of the single-stranded cDNA with 9 PCR cycles. cDNAs in the size range of 600–800 bp were eluted from a preparative agarose gel and sequenced in one full plate (454 GS FLX “Titanium”). Chromatograms from the 454 sequencing (sff files) were extracted using the sff_extract script (available as part of seq_crumbs at http://bioinf.comav.upv.es/). Remaining adapters and trans-spliced leaders were removed using cross_match (Green, http://www.phrap.org), SnowWhite [29,30] and exact matches with custom perl scripts. These steps yielded 1’062’595 reads with an average size of 295 nt and a mode estimated at 330 nt. The full collection of raw 454 reads is available under the study number ERP001719 (sample accession ERS163510) from the European Nucleotide Archive (ENA).

### Preparation and *de*-*novo* assembly of the Illumina reads

For the Illumina RNAseq, nine distinct cDNAs were prepared from regenerating and intact *Hydra*, RNAs were extracted using the QuickPrep Micro mRNA Purification Kit (GE Healthcare) and the polyA mRNA fraction was enriched by performing two rounds of Dynabeads (Invitrogen) polyA selection. The depletion of ribosomal RNA and the overall quality of each fraction was assessed on Bioanalyzer (Agilent). The libraries were prepared by Fasteris SA according to Illumina’s instructions for dir-mRNAseq and mRNAseq protocols. The 3' small RNA adapter was standard Illumina adapter whereas a barcode was inserted in the 5^′^ small RNA adapter. The sequencing was performed on a Genome Analyzer II machine (3 lanes, 76 cycles, 76 nt length including a 4–5 letter barcode). Demultiplexed Illumina reads are available under the study number ERP001719 (sample accessions ERS163501-ERS163509 and ERS163511-ERS163513) from the European Nucleotide Archive (ENA).

The base calling that was performed on the Illumina sequencing images using Ibis [31] yielded 64’433’175 reads of 76 bp. These reads were then processed prior to assemblies as we removed i) the first 5 bp of each read, corresponding to barcodes, ii) the reads that exactly matched the 12 first bp of the Illumina 3’ adapters (not found in any 454 read, thus probably not represented in the *Hydra* transcriptome) and the 3' end of these reads. Then we also removed non-exact matches of adapters by using the -5 and -adapter options of the Novoalign software (http://www.novocraft.com). Finally only reads ≥30bp were kept. After manual inspection, homo-polymers longer than 5 consecutive nucleotides located at 3’ ends were removed. Moreover, reads containing more than 90% of bases with phred quality scores of 5 or less were discarded using the fastq_quality_filter tool (http://hannonlab.cshl.edu/fastx_toolkit/). A total of 53’599’364 (83%) Illumina reads survived these preliminary cleaning steps.

The Illumina reads were then assembled using the Velvet assembler v1.0.18 [32] followed by the Oases transcriptome wrapper v0.1.18 [33]. In total 17 independent assemblies with k-mers from 33 to 65 (with a stepsize of 2) were performed in parallel and pooled together, yielding 455’142 Illumina contigs (Figure 3A). The edges of these contigs were thoroughly cleaned for remaining adapter and trans-spliced leaders sequences by using SeqClean (http://sourceforge.net/projects/seqclean/) followed by removing all edges having an exact TAAG match within their first 15 bp or the reverse complement CTTA within the last 15 bp as these motifs are very common hallmarks of trans-spliced leaders [34,35].

### *De*-*novo* (***dn***) hybrid assemblies of Illumina contigs and 454 reads

Two independent assemblies were performed one using Mira 3.2.1.15 dev [36] (assembly called Mira-*dn*), and another using Newbler v2.6 [18] (Newbler-*dn* assembly), both requesting 50 bp as the minimum overlap parameter and 95% as the minimum identity (Figure 3A). Prior to the hybrid assemblies, Illumina contigs generated by Velvet/Oases were thoroughly cleaned (see above) and were manipulated as follows: 1) Illumina contigs were duplicated, and one bp was removed to the front of each duplicated sequence so as not to produce exact duplicates. The FASTA headers of the duplicates were slightly modified; 2) given the read size limit of the Newbler assembly software (1999 nt as a maximum sequence length for input), longer Illumina contigs were chopped to reach a maximum size of 1999 nt with 1899 nt overlaps (in a manner similar to described in [20] 3) as the Velvet/Oases assembler does not produce quality files, an artificial quality file was then generated for the contigs, attributing strong phred quality scores of 40 to internal bases and weaker scores of 10 to 25 bp edges as edges are more susceptible to contain errors. These combined steps caused the number of Illumina contigs to increase from 455’142 to 1’067’061 and this set was used as input for both Mira and Newbler *de*-*novo* assemblies.

For Mira-*dn*, cleaned 454 reads were input along with Illumina contigs and quality file; for Newbler-*dn* chromatogram files were input directly and a fasta file along with the prepared Illumina contigs and qualities files. In addition, a file containing all adapters and known trans-splice leaders was input as trimming file (-vt option). The outputs of these two assemblies were then joined using a second round of Mira assembly with similar parameters (meta-*dn*). Occasionally, meta-*dn* contained longer sequences than the two original dataset. To avoid unnecessary redundancy, the longest deduced ORFs of a given RNA were selected from the different sets.

### Genome-assisted (ga) assemblies

The ga assembly consisted first on the mapping of extracted and cleaned 454 reads to the most complete version of the *Hydra* genome, i.e. the RP genome assembly (JGI) using the PASA genome annotation tool version r09.01.201 [37-39] with a minimum fraction of the read aligned to a single location of 50% and more than 93% identity (Figure 3B). Overall, 88% of the reads were mapped and validated by PASA against this genome. Illumina reads were then mapped to the genome using TopHat 1.0.1 [40] with default options. The genomic hits retrieved by the TopHat mapper (collected in the BAM file) were input to cufflinks [21] together with the gene transfer format file (.gtf) produced by PASA (as a gtf guide, -g option) and resulted in a single gtf file incorporating information from both the PASA (454 reads) and the TopHat (Illumina reads) processes. The RSEM package [41] was finally used to extract sequences based on the genome coordinates output by cufflinks. Similarly to the *de-novo* assembly process, a second round of assembly was performed using Mira (see above) with this dataset (meta-ga assembly) and the longest open reading frames (ORFs) were retrieved for each sequence.

### Redundancy removal

To keep only the longest unique ORFs, the meta-*dn* and meta-gn sequences were 6-frame translated and the longest ORF of each sequence was selected. Sequences shorter than 50 amino acids were not further considered and the 25 C-terminus of each sequence was trimmed, as the nonsense polypeptide translated after a frame-shift is on average 15 residues long [42]. The proteins sequences were then clustered together using a global identity threshold of 95% and a gap penalty of 20 using usearch [43] to retrieve the longest sequence of each cluster. Remaining sequences were aligned on themselves using the Basic Local Alignment Search Tool (Blast) [44] without using the low complexity filter. The following procedure was used to characterize and discard the sequences (matches) similar but shorter than the query i) alignments smaller than 25 aa and self-reported matches were ignored, ii) the query needed to be bigger than the match, iii) the pairwise identity of the best alignment block per match needed to be >95%, iv) there were no gaps in the alignment (to retain splice variants), v) the alignment should span at least 95% of the match, vi) no more than 100 aa from the match were unmatched by the query. Untruncated versions of remaining sequences were retrieved (i.e. containing the 25aa that were removed at the beginning of the procedure).

### Removal of known contaminants in the RNAseq only and predicted-only datasets

To confirm the *Hydra* origin of the 10’741 genome-unpredicted “RNAseq only” and the 11’582 genome-predicted only sequences, we tested these sequences sequentially in successive BlastN+ analyses, first against the sequences of common *Hydra* contaminants (*Artemia*, *Curvibacter*), then against *Hydra* genomes. The sequences found in one database were not realigned to the next database. Using this method, we removed from the 10’741 RNAseq only sequences, 144 contigs not attributed to *Hydra*, composed of 114 contigs of very low complexity (i.e. stretches of repetitive elements), 21 transcripts corresponding to known *Artemia franciscana* sequences (*Hydra* diet in the lab), and nine others matching the genome of the β-proteobacteria *Curvibacter*, a known *Hydra* symbiont. Concerning the 11’582 genome-predicted only sequences, we removed 312 sequences considered as contaminants, 247 from *Curvibacter*, and 65 as low-complexity sequences. The resulting 10’597 RNAseq only and 11’270 predicted-only sequences were used for further analyses.

### Sequence annotation

The *Hydra*-bo, *AEP*-*454*, pred-CA, and pred-RP transcriptomes were aligned by BlastX+ with an E-value threshold of 10^-10^ to the full Swissprot and the cnidaria-UniProt databases (releases 2013_01). Annotations of domains and protein families were performed on deduced protein sequences with Pfam 26.0 [45] and Panther 8.0 [46]. Detailed results are presented in Additional file 2.

### Production of a comprehensive *Hydra* transcriptome containing RNAseq and genome-predicted sequences (*Hydra*-meta dataset)

Transcripts arising from the same locus (and overlapping) were merged together to form one single transcript. The two distinct *Hydra magnipapillata* genome assemblies currently available [3], i.e. the Celera Assembler assembly (CA, GenBank accession number ABRM00000000) and the Ringer-Phrap assembly (RP, GenBank accession number ACZU00000000) are associated with two predicted sets of transcripts referred to as “pred-CA” and “pred-RP” here: the gene model set associated with the CA genome contains 17’741 transcripts and the RP-derived set 32’338 transcripts. We produced a non-redundant set by pooling the RNA sequences of the *Hydra*-bo (48’909 sequences), pred-CA (17’741), and pred-RP (32’338) datasets, aligned them to the *Hydra* genomes by PASA. Sequences were first aligned to the RP genome, then, the sequences not suitably aligned were realigned onto the CA genome. Sequences not properly aligning to either genome were also retrieved as they might represent true transcripts originating from currently unavailable portions of the genome. All sequences represented an intermediate dataset of 70’368 transcripts. Overall, 59’316 sequences were validated on one or the other genome (arising from 48’217 different loci), 10’208 were found in the genome but failed alignment validation due to the PASA options used (see above), and 844 sequences did not align to the genome at all. The longest ORF of each transcript was extracted, and a final round of global clustering was performed on the protein sequences using usearch with a minimum similarity threshold of 95% and strong gap penalties to retain most splice variants (i.e. using the options -id 0.95 -iddef 2 -gapopen 20). The final set contains 57’611 protein sequences (Figure 4A). The characteristics of the datasets are detailed in Additional file 1: Table S1. The *Hydra*-bo assembly is deposited at the European Nucleotide Archive (ENA) under the project number PRJEB445 and with accession numbers from HAAC01000001 - HAAC01045269.

### Computation of the Redundancy Index (RI)

Megablast was parameterized such as only matches with more than 98% identity and an E-value ≤10^-30^, were retrieved. De facto, a limit of maximum 250 alignments per query is specified by the default options. Importantly, using coding sequences rather than full length sequences strongly reduced biases caused by vector contaminants, repetitive sequences or post-translational modification such as the addition of trans-splice leaders. In turn, the soft filtering allows masking low complexity stretches during the search phase, while using them in the scoring phase. Without the later, too many hits would fall below the 98% identity threshold due to masked (low complexity) stretches counted as mismatches.

### Comparison of the sizes of the coding lengths to the sizes of the coding sequences in the pred-CA transcriptome

Using megablast, the pred-CA coding transcripts were aligned to the each of the other coding transcriptomes using a threshold of 98% sequence identity without low complexity filter. The first hit was considered as representing the same coding sequence than the query if the length of the alignment exceeded 100 nt. The length of the tested contigs and corresponding pred-CA contigs were compared using custom Perl scripts. Similarly, each pred-CA sequence was considered has having no representative in the tested dataset for alignment spanning less than 100nt or having less than 98% identity (Figure 5B, top numbers).

### Saturation analysis

Fractions of the total number of Illumina reads (53’599’364 reads) ranging from 1% to 100% were selected by generating the required amount of random numbers lying between 1 and 53’599’364 using R (sampling without replacement). Corresponding reads were then fetched from the original dataset using a custom Perl script. Subsets of reads were realigned to the *Hydra*-meta dataset using Bowtie 0.12.09 [47] with default options.

### Analysis of Reciprocal Best Hits (RBHs) and sources of proteomes

The *Hydra* proteomes deduced from predicted-only and RNAseq only sequences were used as input for BlastP+ using a E-value threshold of 10^-8^, with soft masking as suggested by [23]. Relations retained as Reciprocal Best Hit (RBHs) fulfilled two criteria: 1) best score between a given query and the different hits, 2) best score between a given hit and the different queries. RBH analyses were performed on a reference dataset containing representative proteomes selected for their completeness and limited redundancy. Proteomes were obtained from UniProtKB [48] for *Branchiostoma floridae* (*Brafl*), *Caenorhabditis elegans* (*Caeel*), *Capsaspora owczarzaki* (*Capow*), *Drosophila melanogaster* (*Drome*), *Gallus gallus* (*Galga*), *Macaca mulatta* (*Macmu*), *Monosiga brevicollis* (*Monbr*), *Nematostella vectensis* (*Nemve*), *Saccharomyces cerevisiae* (*Sacce*), *Salpingoeca rosetta* (*Salro*), *Tribolium castaneum* (*Trica*), *Trichinella spiralis* (*Trisp*), *Xenopus tropicalis* (*Xentr*); from Swissprot for *Homo sapiens* (release 2011_07, 20’231 proteins); from the DOE Joint Genome Institute [49] for *Amphimedon queenslandica* v1.0 (*Ampqu*), *Capitella teleta* v1.0 (*Capte*), *Ciona intestinalis* v2.0 (*Cioin*), *Saccoglossus kowalevskii* v1.3 (*Sacko*-*JGI*); from the NCBI Reference Sequence collection (RefSeq [50]) for *Saccoglossus kowalevskii* (Sk-RefSeq), release 53; from the Okinawa Institute of Science and Technology (OIST) for *Acropora digitifera* (*Acrdi*) *v1*.*0*, as described in [5]. Finally the *Clytia haemispherica* (*Clyhe*) dataset was obtained by merging all *Clytia* EST sequences available on Compagen [27] and Genebank (2011/11/01); after conceptual translation, the redundant sequences where removed using the usearch software [43].

### RT-PCR amplification of RNAseq only and genome-predicted only transcripts

RNA was extracted using the RNeasy mini kit (Qiagen, #74106) using a mixture of 180 animals from the *Hydra vulgaris* Basel strain (budding, regeneration time points between 20 minutes and 32 hours, animal starved during one week). DNase digestion was performed using a RNAse-free DNAse set (Qiagen, #79254) and RNA was precipitated. After resuspension, reverse transcription was performed using the Superscript III reverse transcriptase (Life Technologies, #18080044) with random hexamers. All PCRs were performed during 40 cycles using the TopTaq polymerase (Qiagen, #200201). All primer sequences are available in the Additional file 4. For detecting the *Hydra*-human orthologs a similar procedure was applied except that no DNAse treatment was performed and sequences were amplified for 34 cycles.

### Phylogenetic analyses

For each protein of interest, sets of similar sequences were retrieved after BlastP+ searches performed on NCBI and Uniprot databases. Phylogenetic analyses were performed on sequences aligned with Muscle [51] and trees were obtained with the PhyML 3.0 program [52] with the following options: LG model of amino acids substitution, discrete gamma model, number of categories: 8, gamma shape parameter: 1, BioNJ as initial tree, tree topology fixed, 500 bootstraps. The 5 letters species code used here is from Uniprot. The fasta files are available on request.

## Competing interests

 BOth authors declare that they have no competing interest.

## Authors’ contributions

BG and YW designed the experiment, analyzed the results, and wrote the manuscript; YW performed the transcriptome assembly. Both authors read and approved the final manuscript.

## Supplementary Material

Additional file 1 Contains 5 figures and 1 table. Click here for file

Additional file 2 Tables containing gene families characterized by BlastX+, PANTHER and PFAM in the various Hydra transcriptomes (Excel file). Click here for file

Additional file 3 **Tables containing the primers and the criteria used to test the expression of a subset of *Hydra*-bo RNA-seq only and predicted-only genes with a summary of the results (Excel file).** Click here for file

Additional file 4 Table containing the sequences and annotations of the 170 novel evolutionarily-conserved genes identified in Hydr-bo but not predicted in the genome (Excel file). Click here for file
