# Supplementary material for: RNAseq versus genome-predicted transcriptomes: a large population of novel transcripts identified in an Illumina-454 Hydra transcriptome
Source: BMC Genomics. 2013 Mar 25;14:204. doi: 10.1186/1471-2164-14-204 (PMC3764976; doi:10.1186/1471-2164-14-204)
Supplement: Additional file 1 — Contains 5 figures and 1 table. [file 1471-2164-14-204-S1.pdf]

RNAseq versus genome-predicted transcriptomes: A large population of novel transcripts identified in an Illumina-454 *Hydra* transcriptome

Yvan Wenger[1]
Email: Yvan. Wenger@unige.ch

Brigitte Galliot[1*]
* Corresponding author
Email: Brigitte. Galliot@unige.ch

[1] Department of Genetics and Evolution, Institute of Genetics and Genomics in Geneva (iGE3), University of Geneva, Geneva, Switzerland

ADDITONAL FILE 1

Table S1: Access to the various *Hydra* RNAseq datasets 2
Figure S1: Effect of duplication of the illumina contigs set on the hybrid transcriptome assembly 3
Figure S2: Characteristics of the *Hydra*-bo RNA-seq, genome-predicted and *Hydra*-meta datasets. 4
Figure S3: Similar conservation of the *Hydra*-bo, genome-predicted and *Hydra*-meta datasets across evolution. 5
Figure S4: Phylogenetic analyses of 14 genome-unpredicted *Hydra* proteins identified in the *Hydra*-bo transcriptome confirm orthology assignment by the RBH method. 7
Figure S5: RT-PCR analyses confirm the expression of most genome-unpredicted RNAseq-only transcripts (Classes I, II, III). 8
Additional References 8

Dataset name	Dataset specifications	Dataset size	Access to data
***Hydra*-dn (de novo)**	454 and Illumina reads assembly	**37’523** transcripts	hydra-dn dataset, 37’523 sequences
***Hydra*-ga (genome assisted)**	**Genome assisted assembly:** 454 + *Hydra* RP genome + Illumina reads assembly	**33’422** transcripts	hydra-ga dataset, 33'422 sequences
***Hydra*-bo (best of)**	**RNA-seq reads:** De-novo + genome-assisted (ga)-assemblies	**48’909** sequences **45’269** > 200 nt	Complete hydra-bo dataset, 48'909 sequences<br>**European Nucleotide Archive (ENA)** HAAC01000001-HAAC01045269
***Hydra*-meta**	*Hydra*-bo pooled with the genome-predicted datasets and realigned to the RP and CA genomes	**57’611** sequences with ORFs	**Nucleotide sequences:** http://genev.unige.ch/system/supp_data/BMCGenomics2013/hydra_meta.fasta<br>**Deduced protein sequences:** http://genev.unige.ch/system/supp_data/BMCGenomics2013/hydra_meta_proteins.fasta

Table S1: Access to the various *Hydra* RNAseq datasets

All datasets are available at: http://genev.unige.ch/system/supp_data/BMCGenomics2013/index.html

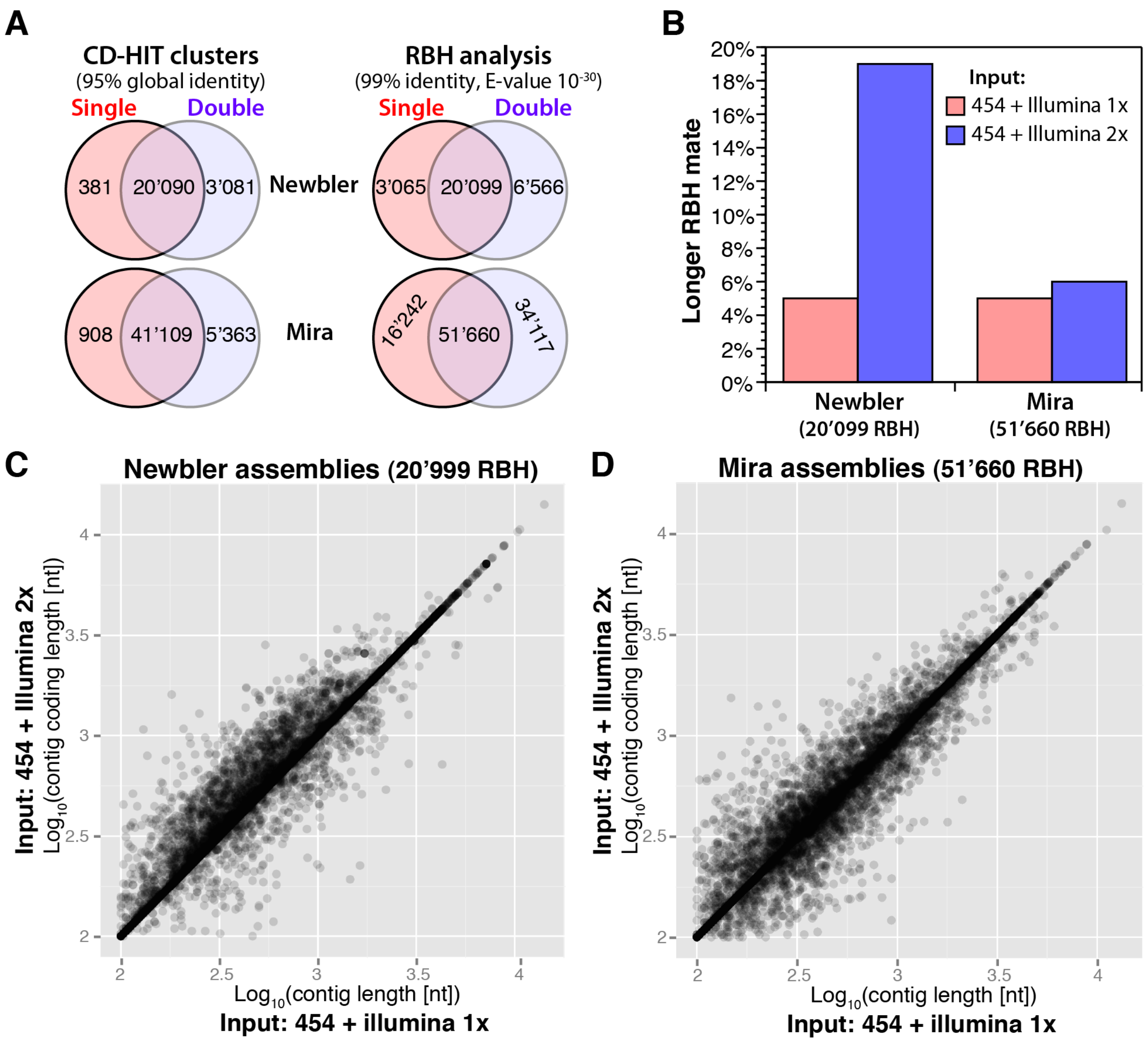

Figure S1: Effect of duplication of the illumina contigs set on the hybrid transcriptome assembly.

To asses the effect of manipulating the illumina contigs produced by Velvet/Oases, the subsequent step of merging with 454 reads were performed with and without doubling the illumina contigs. Note that the two conditions with doubled datasets correspond to the assembly of mira *de-novo* and newbler *de-novo* shown in **Figure 3A**. **A)** Cluster analyses performed on deduced coding nucleotides ORFs using either the CD-HIT-est [1] (global alignment, one or more sequences retrieved per cluster) or RBH (local alignment, only the two best mate pair, one of each dataset retrieved by cluster) methods. Overall, a majority of the contigs generated has representatives in both single and doubled sets as shown with the CD-HIT clustering. Also, assemblies with doubled illumina contigs generate higher numbers of unique contigs. As RBH provides with a list of best sequences pairs (mates), these mate pairs were used in other panels of this figure. **B)** Count of coding nucleotide ORFs that are longer in the RBH mate pairs identified. Mira assemblies are mildly affected with ~10% of mate pairs that are longer in one or the other assembly (single versus doubled illumina input). In contrast, up to 19% of newbler contigs generated with the doubled dataset are longer than in the non-doubled dataset whereas ~5% are longer with the non-doubled dataset. The fraction of sequences of identical coding lengths is 77% and 89% for newbler and mira RBHs mate pairs. **C, D)** Detailed view of the coding sizes among RBH mate pairs retrieved for newbler and mira assemblies, respectively.

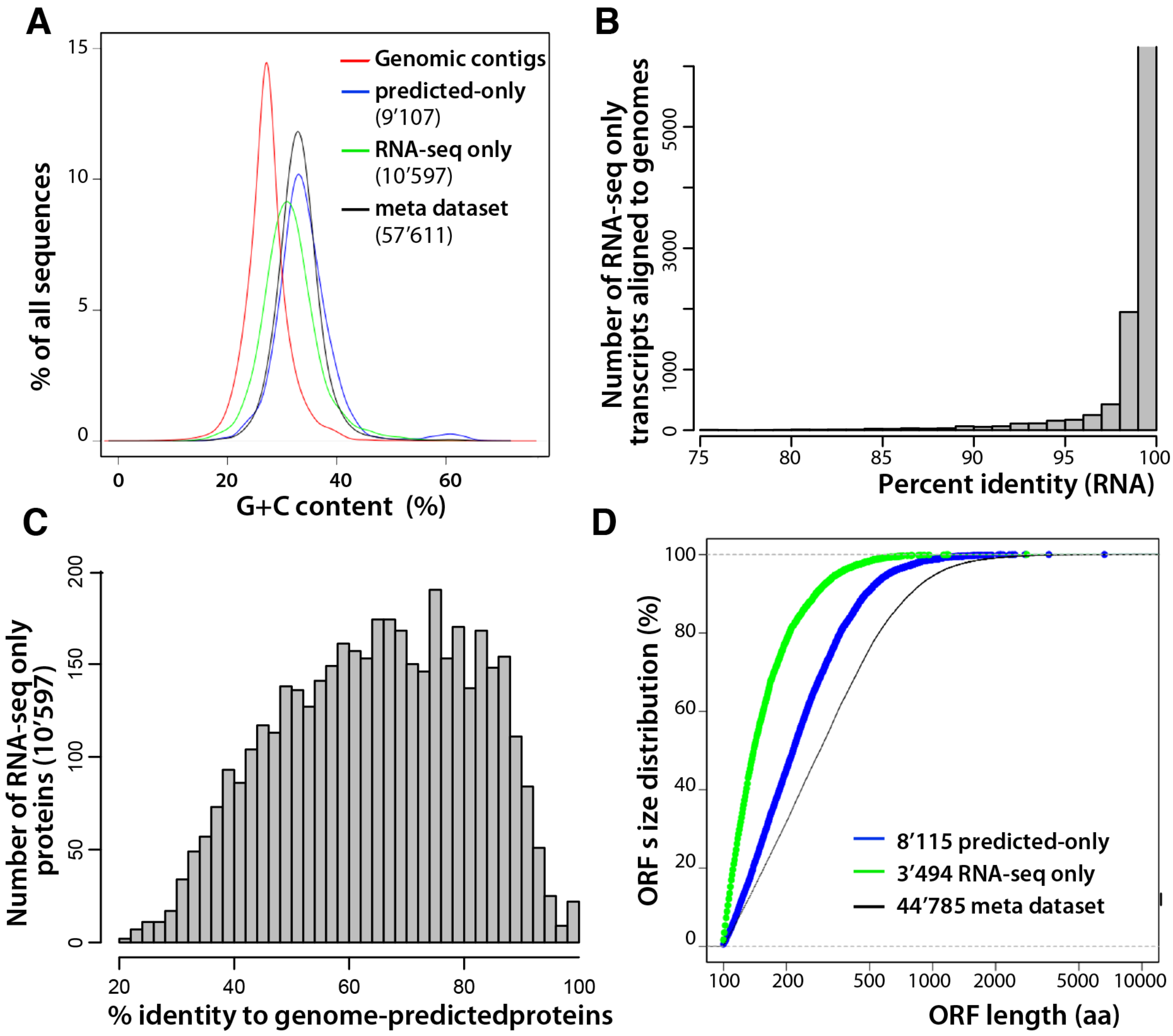

Figure S2: Characteristics of the *Hydra*-bo RNA-seq, genome-predicted and *Hydra*-meta datasets.

A) Similar GC content in RNA-seq only (green), predicted-only (blue), genomic (red) and meta-dataset (black) sequences. **B)** Distribution of the 9'305 RNA-seq only transcripts according to their identity with the *Hydra* genomic sequences. Percent identity was obtained from Blastn matches recorded above 75% identity either on the CA or on the RP genomes. **C)** 4'103 deduced proteins from the the 10'597 RNA-seq only transcripts show a significant similarity with the predicted proteins (pred-CA and pred-RP) as deduced from BLASTx alignment preformed with a E-value lower than 10^{-10}. **D)** Cumulative ORF size distribution of the RNA-seq only (green), predicted-only (blue) and meta dataset (black). Only proteins longer than 100 aa were considered here (RNA-seq only: 3'537, predicted-only: 9'107, meta dataset: 44'785).

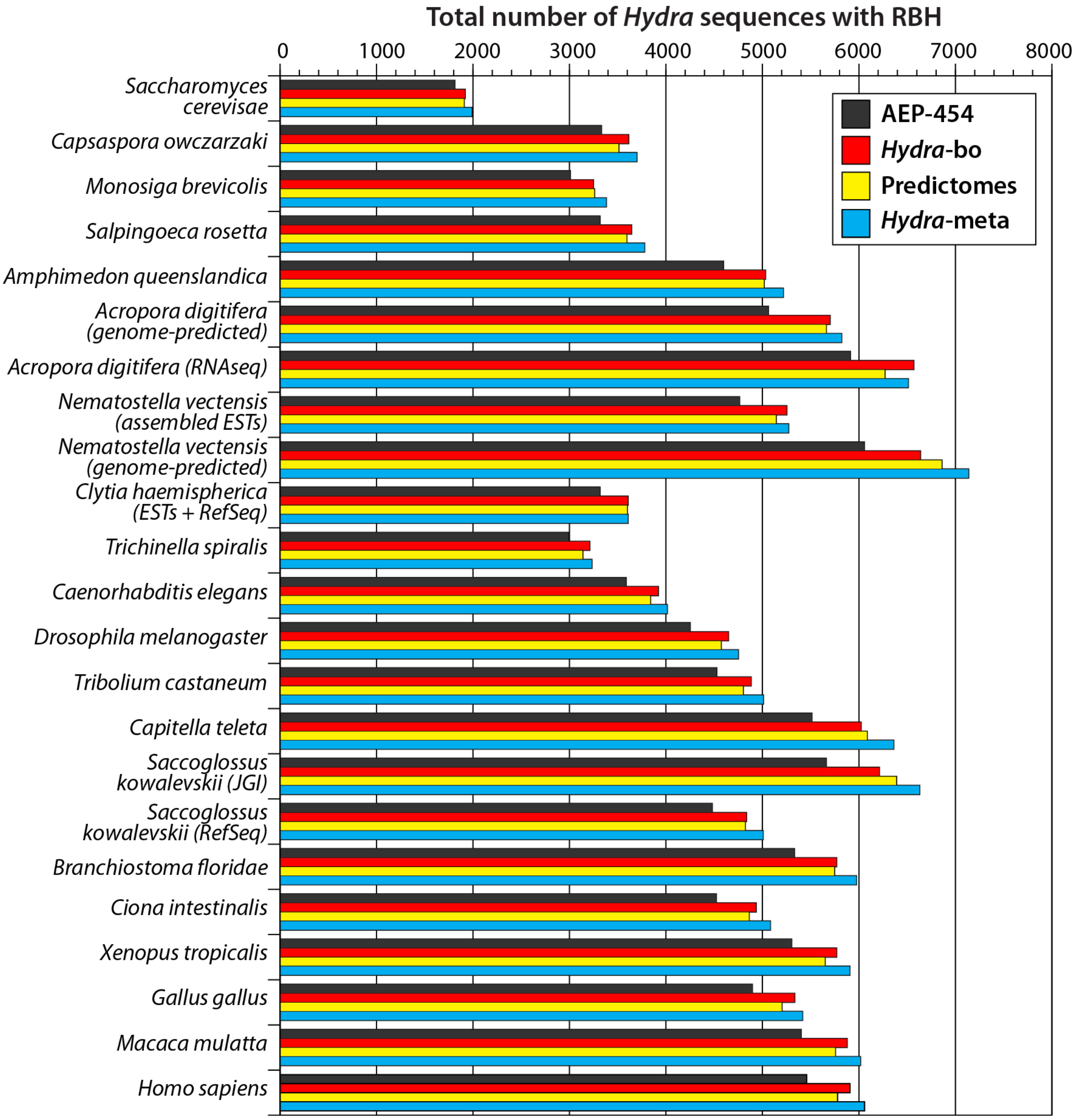

Figure S3: Similar conservation of the *Hydra*-bo, genome-predicted and *Hydra*-meta datasets across evolution.

Reciprocal best hits (RBHs) were retrieved from the three datasets, *Hydra*-bo (red bars), genome-predicted (yellow bars) and *Hydra*-meta (blue bars) tested on the proteomes of the species indicated above. Note the similar number of orthologs detected in the *Hydra*-bo and genome-predicted datasets and the slightly increased number of orthologs detected in the *Hydra*-meta dataset.

1. Phosphoglycolate phosphatase (PGP)

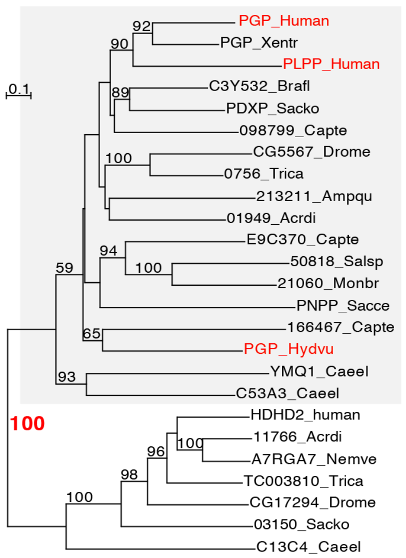

2. Sperm flagellar protein 2 (SPEF2)

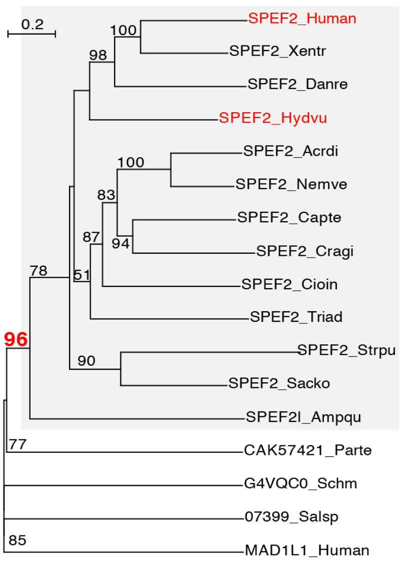

3. Fasciculation and elongation protein zeta-2 (FEZ2)

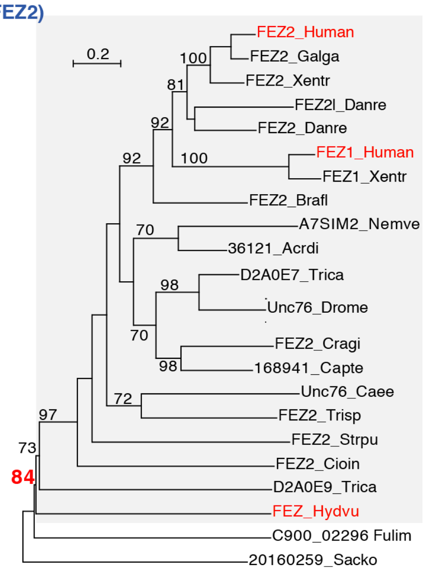

4. Protein MMS22-like (MMS22L)

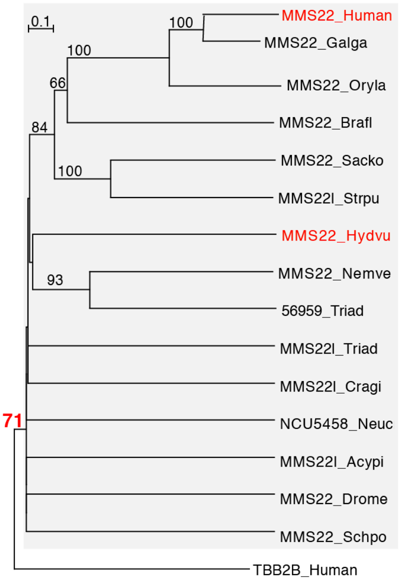

5. NjmuK

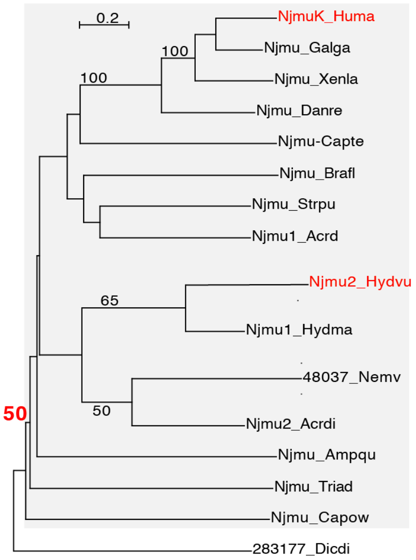

6. Dolichol kinase (DOLK)

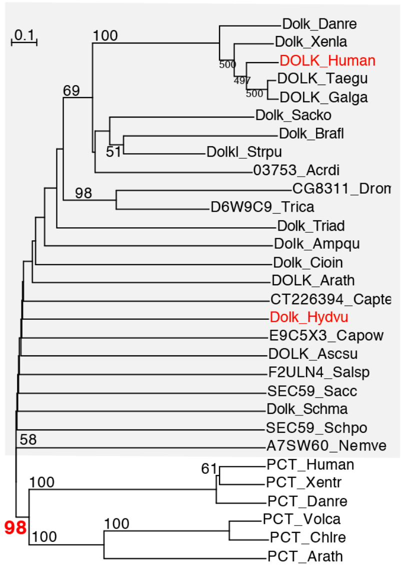

7. FYVE and coiled-coil domain-containing protein 1 (FYCO1)

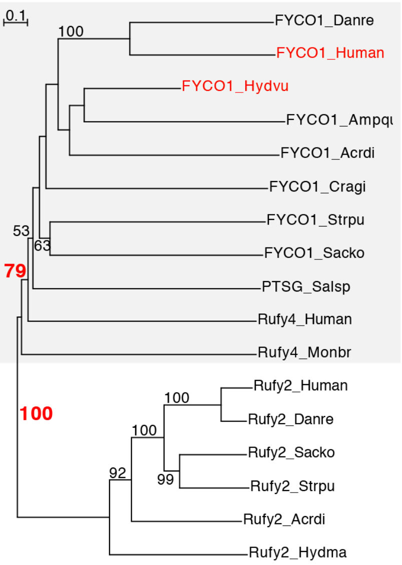

8. Folylpolyglutamate synthase mitochondrial (FPGS or FOLC)

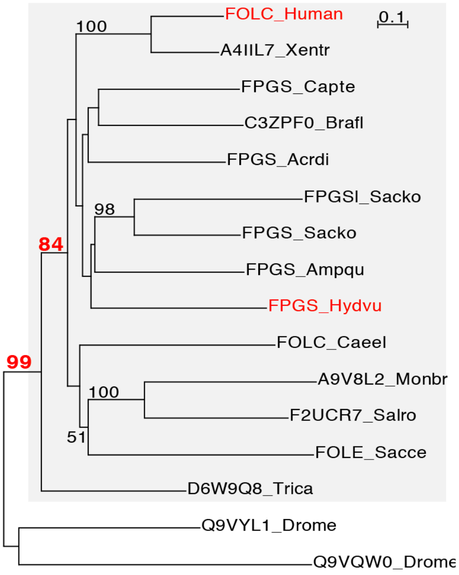

9. Hermansky-Pudlak syndrome 5 (HPS5)

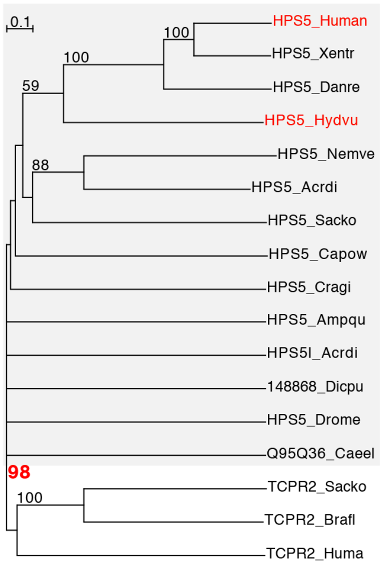

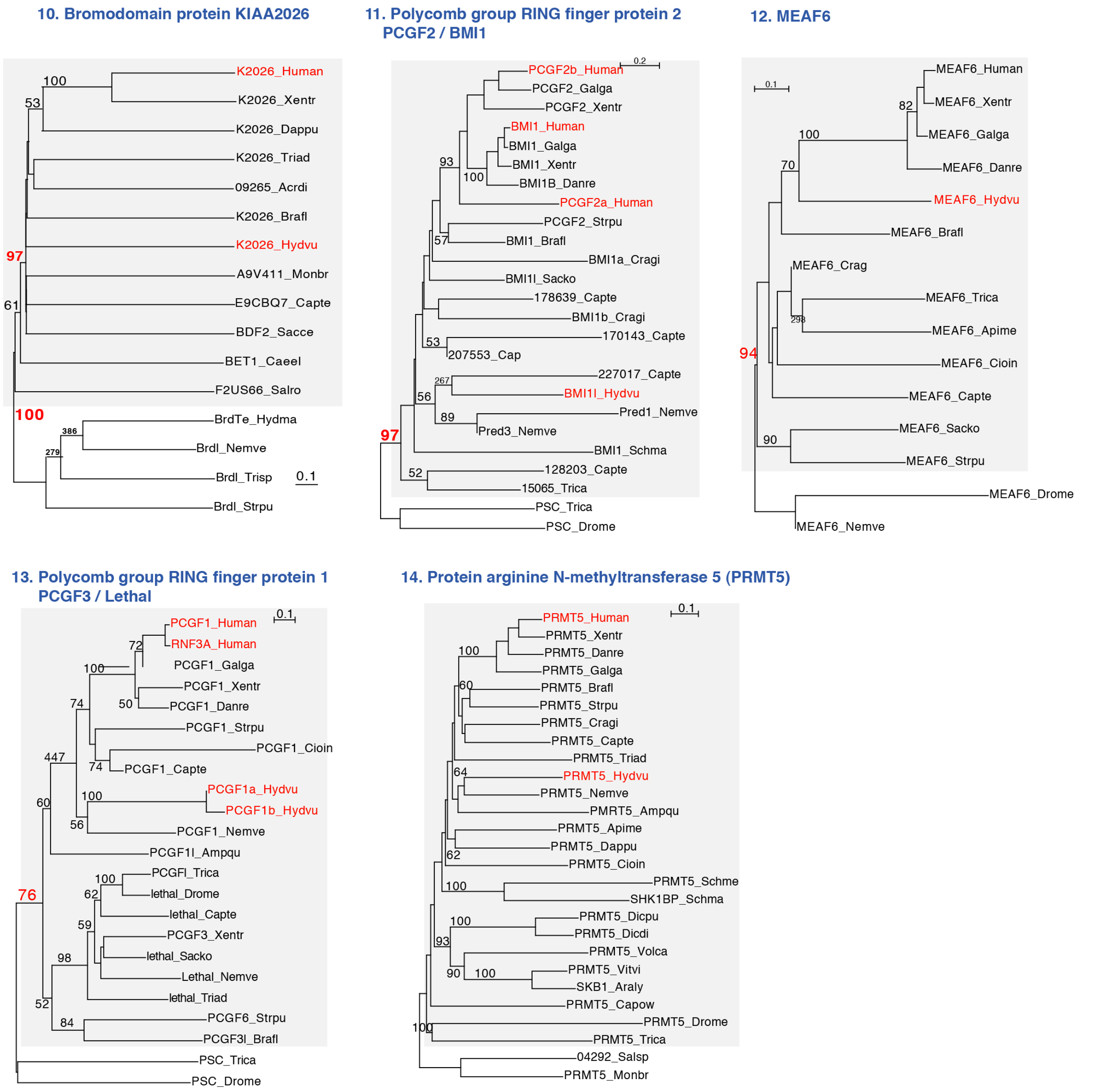

Figure S4: Phylogenetic analyses of 14 genome-unpredicted *Hydra* proteins identified in the *Hydra*-bo transcriptome confirm orthology assignment by the RBH method.

Phylogenetic analyses were performed on sequences aligned with Muscle [2] and trees were obtained with the PhyML 3.0 [3] program (see Methods in the main text). The 5 letters species code used here is from Uniprot: *Acrdi: Acropora digitifera* (Cnidaria Anthozoa, coral); *Acypi: Acyrthosiphon pisum* (Arthropoda Insecta, pea aphid); *Ampqu: Amphimedon queenslandica* (Porifer, sponge); *Apime: Apis mellifera* (Arthropoda, honeybee); *Brafl: Branchiostoma floridae* (Cephalochordata, amphioxus); *Caeel: Caenorhabditis elegans* (Nematoda, roundworm); *Capow: Capsaspora owczarzaki* (Filasterea, amoeboid unicellular symbiont); *Capte: Capitella teleta* (Annelida, polychaete worm); *Cragi: Crassostrea gigas* (Mollusca, oyster), *Cioin: Ciona intestinalis* (Urochordata, sea squirt); *Clyhe: Clytia hemispherica* (Cnidaria Hydrozoa, jellyfish); *Danre: Danio rerio* (Actinopterygii, zebrafish); *Dappu: Daphnia pulex* (Arthropoda crustacea, waterflea)*; Dicdi, Dicpu: Dictyostelium discoideum, D. purpureum* (Amoebozoa, slime mold)*; Drome: Drosophila melanogaster* (Arthropoda Insecta, fruit fly); *Galga: Gallus gallus* (Aves, chick); *Human: Homo sapiens* (Primates); *Hydma, Hydvu: Hydra magnipapillata, H. vulgaris* (Cnidaria Hydrozoa, freshwater polyp); *Nemve: Nematostella vectensis* (Cnidaria Anthozoa, sea anemone); *Monbr: Monosiga brevicollis* (Filozoa, choanoflagellate); *Neucr: Neurospora crasse* (Ascomycota); *Oryla: Oryzias latipes* (Actinopterygii, medaka); *Sacce: Saccharomyces cerevisiae* (Ascomycota, budding yeast); *Sacko: Saccoglossus kowalevskii* (Hemichordata, acorn worm); *Salsp, Salro: Salpingoeca sp, S. rosetta* (Filozoa, choanoflagellate); *Schma: Schistosoma mansoni* (Platyhelminthe, trematode); *Schme: Schmidtea mediterranea* (Platyhelminthe, planaria); *Schpo: Schizosaccharomyces pombe* (Ascomycota, fission yeast); *Strpu: Strongylocentrotus purpuratus* (Echinodermata, sea urchin); *Triad: Trichoplax adhaerens* (Placozoa); *Trica: Tribolium castaneum* (Arthropoda Insecta, beetle); *Trisp: Trichinella spiralis* (Nematoda, parasite worm); *Xentr: Xenopus tropicalis* (Amphibia, tropical clawed frog). Fasta files available on request.

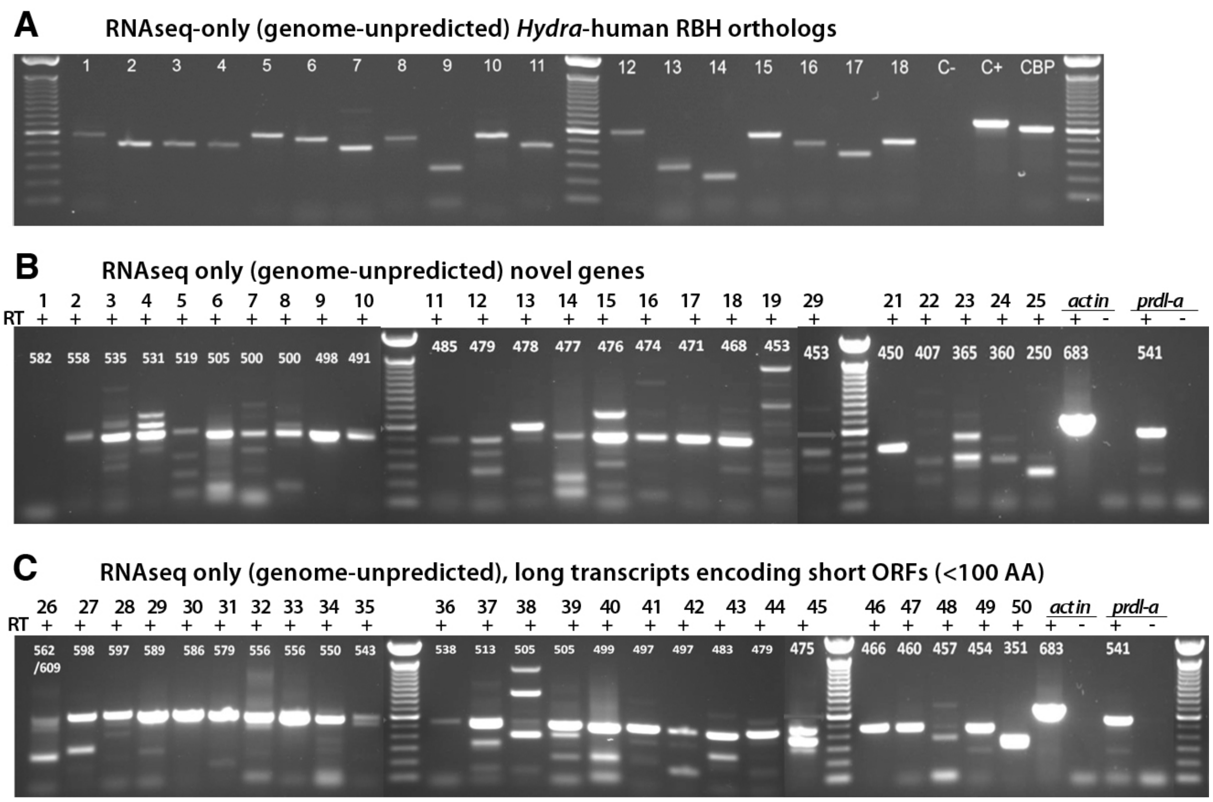

Figure S5: RT-PCR analyses confirm the expression of most genome-unpredicted RNAseq-only transcripts (Classes I, II, III).

A) RT-PCR detection of 18 evolutionarily-conserved gene families that were not predicted from the genome but could be identified among the *Hydra*-bo RNAseq transcripts (Class I). Of the 18 transcripts tested, all were positive: **1.-** Phosphoglycolate phosphatase (PGP), **2.-** Sperm flagellar protein 2 (SPEF2), **3.-** Fasciculation and elongation protein zeta-2 (FEZ2), **4.-** Methyl methanesulfonate-sensitivity protein 22-like (MMS22L), **5.-** Bromodomain protein KIAA2026, **6.-** Dolichol kinase (DOLK), **7.-** FYVE and coiled-coil domain-containing protein 1 (FYCO1), **8.-** Hermansky-Pudlak syndrome 5 protein (HPS5), **9.-** Folylpolyglutamate synthase, mitochondrial (FPGS), **10.-** Protein Njmu-R1 (C17orf75), **11.-** GPI mannosyltransferase 2 (PIGV), **12.-** C-Maf-inducing protein (CMIP), **13.-** Mitochondrial import inner membrane translocase subunit Tim9 (TIMM9), **14.-** HAUS augmin-like complex subunit 6 (HAUS6), **15.-** Glomulin (GLMN), **16.-** 28S ribosomal protein S28, mitochondrial (MRPS28), **17.-** Methionine-R-sulfoxide reductase B1 (SEPX1), **18.-** Uncharacterized protein KIAA0513. lanes C+, C-: actin transcripts detected in the presence (+) or absence (-) of cDNA, lane CBP: CREB-binding protein transcripts.

B) RT-PCR detection of 25 genes identified for the first time among the *Hydra*-bo RNAseq only transcripts (Class II). Of the 25 tested genes, expression of 24 is confirmed.

C) RT-PCR detection of 25 genes identified among the *Hydra*-bo RNAseq only dataset as long transcripts (>1'000 nt) encoding short ORFs (< 100 ORFs, Class III). The expression of all 25 tested genes is confirmed. PCRs were performed during 34 (A) or 40 (B, C) cycles.

Additional References

1. Fu L, Niu B, Zhu Z, Wu S, Li W: **CD-HIT: accelerated for clustering the next-generation sequencing data**. *Bioinformatics* 2012, **28**(23):3150-3152.

2. Edgar RC: **MUSCLE: multiple sequence alignment with high accuracy and high throughput**. *Nucleic Acids Res* 2004, **32**(5):1792-1797.

3. Guindon S, Dufayard JF, Lefort V, Anisimova M, Hordijk W, Gascuel O: **New algorithms and methods to estimate maximum-likelihood phylogenies: assessing the performance of PhyML 3.0**. *Syst Biol* 2010, **59**(3):307-321.
